# Di-μ_2_-chlorido-bis­[aqua­(2,2′-bipyridine-4,4′-dicarboxylic acid-κ^2^ *N*,*N*′)(nitrato-κ*O*)copper(II)]

**DOI:** 10.1107/S1600536808028511

**Published:** 2008-11-22

**Authors:** Ke-Fei Han, Hui-Yong Wu, Zhong-Ming Wang, Hong-You Guo

**Affiliations:** a Department of Chemistry, Beijing University of Chemical Technology, Beijing 100029, People’s Republic of China

## Abstract

In the title compound, [Cu_2_Cl_2_(NO_3_)_2_(C_12_H_8_N_2_O_4_)_2_(H_2_O)_2_], which consists of a chloride-bridged Cu^II^ dimer, the Cu atom is in a distorted octa­hedral environment defined by two N atoms from the 2,2′-bipyridine-4,4′-dicarboxylic acid ligand (H_2_bpdca), two bridging chlorido ligands, and two O atoms from an equatorial water mol­ecule and an axial nitrate anion, respectively. The two halves of the dimeric unit are related by an inversion centre at the midpoint between the two Cu atoms. Both carboxylic acid groups in the H_2_bpdca ligand remain protonated, as confirmed by the two sets of C—O bond lengths. The dinuclear mol­ecules are linked into a three-dimensional network *via* inter­molecular hydrogen bonds.

## Related literature

For related literature, see: Aitipamula *et al.* (2002 ▶); Batten & Robson (1998 ▶); Desiraju (2002 ▶); Etter (1990 ▶); Han *et al.* (2007 ▶); Holliday & Mirkin (2001 ▶); Kitagawa *et al.* (2004 ▶); Kumar *et al.* (2006 ▶); Liu *et al.* (2002 ▶); Moulton & Zaworotko (2001 ▶); Ockwig *et al.* (2005 ▶); Schareina *et al.* (2001*a* ▶,*b* ▶); Tynan *et al.* (2004 ▶, 2005 ▶); Wu (2006 ▶); Wu *et al.* (2006 ▶).
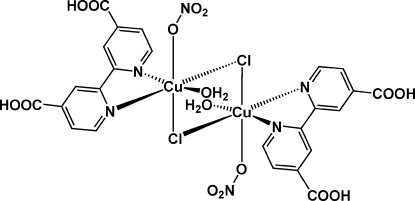


## Experimental

### 

#### Crystal data

[Cu_2_Cl_2_(NO_3_)_2_(C_12_H_8_N_2_O_4_)_2_(H_2_O)_2_]
*M* *_r_* = 846.46
Triclinic, 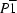

*a* = 6.9500 (7) Å
*b* = 8.1490 (7) Å
*c* = 13.5480 (10) Å
α = 92.315 (2)°
β = 103.384 (4)°
γ = 98.556 (3)°
*V* = 735.91 (11) Å^3^
*Z* = 1
Mo *K*α radiation
μ = 1.72 mm^−1^
*T* = 295 (2) K
0.30 × 0.24 × 0.20 mm

#### Data collection

Rigaku R-AXIS RAPID IP area-detector diffractometer
Absorption correction: multi-scan (*ABSCOR*; Higashi, 1995 ▶) *T*_min_ = 0.627, *T*_max_ = 0.725
5180 measured reflections
3301 independent reflections
3116 reflections with *I* > 2σ(*I*)
*R*_int_ = 0.021

#### Refinement

*R*[*F* ^2^ > 2σ(*F* ^2^)] = 0.035
*wR*(*F* ^2^) = 0.106
*S* = 1.11
3301 reflections
238 parameters
3 restraints
H atoms treated by a mixture of independent and constrained refinement
Δρ_max_ = 0.67 e Å^−3^
Δρ_min_ = −0.72 e Å^−3^

### 

Data collection: *RAPID-AUTO* (Rigaku 2001 ▶); cell refinement: *RAPID-AUTO*; data reduction: *RAPID-AUTO*; program(s) used to solve structure: *SHELXS97* (Sheldrick, 2008 ▶); program(s) used to refine structure: *SHELXL97* (Sheldrick, 2008 ▶); molecular graphics: *SHELXTL* (Sheldrick, 2008 ▶) and *Mercury* (Macrae *et al.*, 2006 ▶); software used to prepare material for publication: *SHELXL97* and *WinGX* (Farrugia, 1999 ▶).

## Supplementary Material

Crystal structure: contains datablocks global, I. DOI: 10.1107/S1600536808028511/rt2020sup1.cif

Structure factors: contains datablocks I. DOI: 10.1107/S1600536808028511/rt2020Isup2.hkl

Additional supplementary materials:  crystallographic information; 3D view; checkCIF report

## Figures and Tables

**Table 1:** Hydrogen-bond geometry (Å, °)

*D*—H⋯*A*	*D*—H	H⋯*A*	*D*⋯*A*	*D*—H⋯*A*
C7—H7⋯O3^i^	0.93	2.45	3.376 (3)	172
C4—H4⋯O3^i^	0.93	2.42	3.346 (3)	179
C2—H2⋯Cl1^ii^	0.93	2.7	3.590 (3)	160
C1—H1⋯Cl1	0.93	2.67	3.258 (3)	122
O5—H5*A*⋯O7^iii^	0.790 (18)	1.798 (19)	2.582 (3)	172 (4)
O2—H2*A*⋯O4^i^	0.77 (4)	1.92 (4)	2.676 (3)	169 (4)
O1—H1*WB*⋯O8^iv^	0.802 (18)	2.10 (2)	2.830 (4)	152 (4)
O1—H1*WA*⋯Cl1^iv^	0.819 (18)	2.48 (2)	3.220 (2)	152 (3)

Symmetry codes: (i) 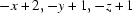; (ii) 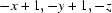; (iii) 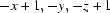; (iv) 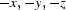.

**Fig. 1.**
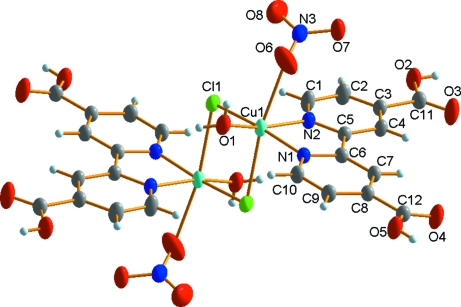
View of a fragment of the title compound, showing 50% probability displacement ellipsoids for non-H atoms. H atoms are shown as small spheres of arbitrary size.

**Fig. 2.**
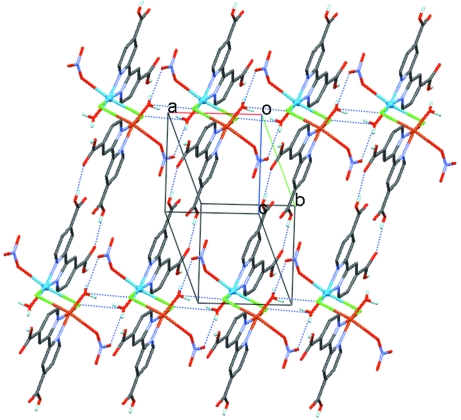
The two-dimensional network formed by hydrogen-bonding interactions (blue dotted lines). For clarity, H atoms attached to C atoms have been omitted.

**Fig. 3.**
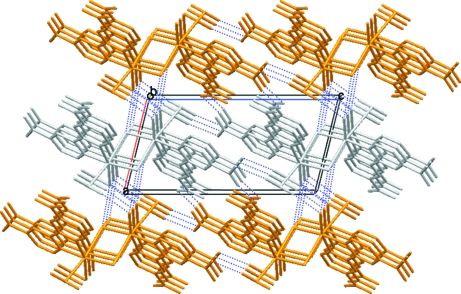
The three-dimensional packing of (I), viewed down the b axis, showing a network structure connected by hydrogen bonds (blue dotted lines). All H atoms have been omitted for clarity.

## supplementary crystallographic information

### Comment

In the field of crystal engineering, based on the metal–ligand coordination interactions, a large number of coordination polymers have been designed and prepared to develop novel functional materials. (For example, Batten & Robson, 1998; Kitagawa *et al.*, 2004; Ockwig *et al.*, 2005). Hydrogen bonding interactions, because of its unique strength and direction, have been widely explored as one of the principal means to control organic molecular assemblies (Desiraju, 2002; Moulton & Zaworotko 2001). As an important synthetic strategy, the combination of both metal–ligand coordination and hydrogen bonding in designing various supramolecular architectures has been extensively used over the past few years (Aitipamula *et al.*, 2002; Holliday & Mirkin, 2001; Kumar *et al.*, 2006; Han *et al.*, 2007). For example, 2,2'-bipyridine-4,4'-dicarboxylic acid (H_2_bpdca), which possesses two N atoms and carboxylic acid groups, has been employed as ligand with the N atoms chelating a metal ion and the carboxylic acid forming either self-complementary hydrogen bonds to neighboring ligands, or coordinating directly to adjacent metal ions following deprotonation (Tynan *et al.*, 2005; Tynan *et al.*, 2004; Liu *et al.*, 2002; Schareina *et al.*, 2001*a*; Schareina *et al.*, 2001*b*; Wu, 2006; Wu *et al.*, 2006). Here we report a copper(II)–H_2_bpdca complex, [Cu_2_(C_12_H_8_N_2_O_4_)_2_Cl_2_(NO_3_)_2_(H_2_O)_2_], (I), with a three-dimensional H-bonding network structure induced by the carboxylic acid groups and water molecules acting as hydrogen-bond donors.

As shown in Fig. 1, the structure of (I) consists of a chloride-bridged Cu(II) dimer, in which the H_2_bpdca ligand remains protonated. The two halves of the dimer unit are related by an inversion centre at the midpoint between the two Cu atoms. Each copper atom has a distorted octahedral geometry, with the equatorial positions utilised by two chelating nitrogen atoms from the H_2_bpdca ligand (average of Cu—N bond length, 2.001 (2) Å), one bridging chlorido ligand (Cu1—Cl1 = 2.2513 (6) Å) and one coordinated water molecule (Cu1—O1 = 1.9828 (21) Å). In the elongated axial direction, one site is occupied by another bridging chloride atom (Cu1—Cl1^i^ = 2.7757 (8) Å, symmetry code: (i), 1 - *x*, -*y*, -*z*), and the other site by an O atom from a nitrate anion (Cu1—O6 = 2.5280 (4) Å). Both bond lengths are longer than those corresponding to usual coordination bonds, indicating a weak coordination. The two pyridyl rings of the H_2_bpdca ligand are slightly twisted, as indicated by its dihedral angle (4.46 (12)°), while the copper-to-copper separation in the dimeric unit is 3.6698 (5) Å. The copper atoms and the bridging chloride atoms occupy the same plane as required by the symmetry, and results in a chloride–chloride separation of 3.4755 (9) Å. The carboxylic group at C11 is almost coplanar with the attached pyridyl ring (dihedral angle *ca* 3.19°), whereas the carboxylic group at C12 is slightly twisted by *ca* 12.31° toward the corresponding pyridyl ring. As expected for protonated carboxylic acids, there are two sets of C—O bond lengths: the carbon to hydroxyl oxygen single bond, which average 1.3095 Å, and the carbon to carbonyl oxygen double bond, which average 1.2013 Å. The coordinated nitrato and chlorido ligands provide the charge balance for the title complex.

The dimeric unit is extended into a three-dimensional network through hydrogen bond interactions (Table 1). Double intermolecular hydrogen bonds (O2—H2A···O4) are formed by means of the double carboxylic acid self-complementary interaction of an adjacent complex, resulting in a centrosymmetric *R*^2^_2_(22) motif (Etter 1990), with the two O3 carbonyl oxygen atoms pointing toward the middle of the ring. Due to its position, the O3 atom can act as a hydrogen bond acceptor from two pyridyl C—H groups (C4—H4···O3 and C7—H7···O3) which further supports the *R*^2^_2_(22) motif. In addition, the coordinated water molecule, O1, is a hydrogen bond donor to the Cl1 atom on adjacent complexes (O1—H1WA···Cl1). These intermolecular hydrogen bonds result in a two-dimensional structure (Fig. 2). Further hydrogen bonding between the non-coordinated O7 atom of nitrate anion and a carboxylic acid group (O5—H5A···O7), and between the O8 atom of same nitrate anion and water molecule of an adjacent complex (O1—H1WB···O8), links the two-dimensional frame into a three-dimensional network (Fig. 3).

### Experimental

A mixture of CuCl_2_.2H_2_O (0.0170 g, 0.1 mmol), H_2_bpdca (0.0244 g, 0.1 mmol) in the molar ratio 1:1, was placed in a 25 ml Teflon-lined digestion bomb with 4 ml distilled water and 1 ml concentrated HNO_3_. The sealed vessel was heated to 473 K for 10 h and then slowly cooled to room temperature (3 K h^-1^). The resulting blue solution was allowed to stand in air at room temperature for one month, yielding blue crystals in 46% yield based on H_2_bpdca. IR spectroscopic analysis (solid KBr disc, ν, cm^-1^): 3410.3(*s*), 3179.0(*m*) 1731.2 (*vs*), 1694.4(*m*), 1625.5 (*m*), 1560.5(*m*), 1403.8 (*vs*), 1382.1 (*vs*), 1235.3 (*m*), 1209.8 (*s*), 1036.3 (*w*), 824.0 (*w*), 660.6 (*s*).

### Refinement

All H atoms attached to C atoms were placed in geometrically idealized positions, with C_sp3_—H = 0.97 Å and C_sp2_—H = 0.93 Å, and constrained to ride on their parent atoms, with *U*_iso_(H) = 1.2*U*_eq_(C). The remaining H atoms attached to O atoms were located in difference Fourier maps and their positional parameters were refined with O—H distances restrained to 0.77 (4)–0.82 (2) Å with *U*_iso_(H) = 1.5*U*_eq_(O).

### Crystal data

**Table:**

[Cu_2_Cl_2_(NO_3_)_2_(C_12_H_8_N_2_O_4_)_2_(H_2_O)_2_]	*Z* = 1
*M**_r_* = 846.46	*F*_000_ = 426
Triclinic, *P*1	*D*_x_ = 1.91 Mg m^−^^3^
Hall symbol: -P 1	Mo *K*α radiation λ = 0.71073 Å
*a* = 6.9500 (7) Å	Cell parameters from 6664 reflections
*b* = 8.1490 (7) Å	θ = 1.6–27.5º
*c* = 13.5480 (10) Å	µ = 1.72 mm^−^^1^
α = 92.315 (2)º	*T* = 295 (2) K
β = 103.384 (4)º	Block, blue
γ = 98.556 (3)º	0.30 × 0.24 × 0.20 mm
*V* = 735.91 (11) Å^3^	

### Data collection

**Table:**

Rigaku R-AXIS RAPID IP area-detector diffractometer	*R*_int_ = 0.021
ω oscillation scans	θ_max_ = 27.5º
Absorption correction: multi-scan(ABSCOR; Higashi, 1995)	θ_min_ = 1.6º
*T*_min_ = 0.627, *T*_max_ = 0.725	*h* = −9→9
5180 measured reflections	*k* = −10→10
3301 independent reflections	*l* = −17→17
3116 reflections with *I* > 2σ(*I*)	

### Refinement

**Table:**

Refinement on *F*^2^	3 restraints
Least-squares matrix: full	H atoms treated by a mixture of independent and constrained refinement
*R*[*F*^2^ > 2σ(*F*^2^)] = 0.035	*w* = 1/[σ^2^(*F*_o_^2^) + (0.0591*P*)^2^ + 0.6991*P*] where *P* = (*F*_o_^2^ + 2*F*_c_^2^)/3
*wR*(*F*^2^) = 0.106	(Δ/σ)_max_ < 0.001
*S* = 1.11	Δρ_max_ = 0.67 e Å^−^^3^
3301 reflections	Δρ_min_ = −0.72 e Å^−^^3^
238 parameters	Extinction correction: none

### Special details

**Table:**

Geometry. All e.s.d.'s (except the e.s.d. in the dihedral angle between two l.s. planes) are estimated using the full covariance matrix. The cell e.s.d.'s are taken into account individually in the estimation of e.s.d.'s in distances, angles and torsion angles; correlations between e.s.d.'s in cell parameters are only used when they are defined by crystal symmetry. An approximate (isotropic) treatment of cell e.s.d.'s is used for estimating e.s.d.'s involving l.s. planes.

### Fractional atomic coordinates and isotropic or equivalent isotropic displacement parameters (Å^2^)

**Table:**

	*x*	*y*	*z*	*U*_iso_*/*U*_eq_	
O6	0.1325 (5)	0.1681 (3)	0.1741 (2)	0.0691 (8)	
Cu1	0.37742 (4)	0.02464 (4)	0.10473 (2)	0.02764 (12)	
Cl1	0.30801 (9)	0.10642 (8)	−0.05463 (4)	0.03186 (15)	
N1	0.4725 (3)	−0.0507 (2)	0.24369 (15)	0.0250 (4)	
N2	0.5689 (3)	0.2299 (3)	0.16874 (16)	0.0274 (4)	
N3	0.1423 (4)	0.3230 (4)	0.1725 (2)	0.0465 (6)	
O1	0.1477 (3)	−0.1609 (3)	0.07198 (16)	0.0380 (4)	
O2	0.9538 (3)	0.7885 (2)	0.28428 (16)	0.0396 (5)	
O3	1.0190 (4)	0.6606 (3)	0.42733 (17)	0.0536 (6)	
O4	0.8547 (3)	−0.0671 (3)	0.60227 (16)	0.0454 (5)	
O5	0.6480 (3)	−0.3083 (2)	0.57019 (15)	0.0395 (5)	
O7	0.2266 (4)	0.4133 (3)	0.25273 (16)	0.0443 (5)	
O8	0.0715 (7)	0.3831 (6)	0.0958 (2)	0.1053 (15)	
C1	0.6048 (5)	0.3690 (3)	0.1234 (2)	0.0368 (6)	
H1	0.546	0.371	0.0545	0.044*	
C2	0.7267 (5)	0.5113 (3)	0.1753 (2)	0.0369 (6)	
H2	0.7505	0.607	0.1419	0.044*	
C3	0.8119 (4)	0.5075 (3)	0.27753 (19)	0.0269 (5)	
C4	0.7771 (4)	0.3630 (3)	0.32546 (18)	0.0264 (5)	
H4	0.8347	0.3585	0.3943	0.032*	
C5	0.6542 (3)	0.2250 (3)	0.26844 (17)	0.0238 (4)	
C6	0.6057 (3)	0.0642 (3)	0.31031 (18)	0.0241 (4)	
C7	0.6879 (4)	0.0294 (3)	0.40842 (18)	0.0256 (5)	
H7	0.7793	0.1096	0.4532	0.031*	
C8	0.6319 (4)	−0.1272 (3)	0.43924 (18)	0.0247 (4)	
C9	0.4958 (4)	−0.2451 (3)	0.37088 (19)	0.0289 (5)	
H9	0.4564	−0.3507	0.3903	0.035*	
C10	0.4201 (4)	−0.2024 (3)	0.27330 (19)	0.0287 (5)	
H10	0.3303	−0.2814	0.2269	0.034*	
C11	0.9409 (4)	0.6586 (3)	0.3387 (2)	0.0298 (5)	
C12	0.7236 (4)	−0.1641 (3)	0.54572 (19)	0.0276 (5)	
H1WA	0.050 (4)	−0.139 (5)	0.090 (3)	0.041*	
H1WB	0.113 (5)	−0.204 (4)	0.0152 (17)	0.041*	
H2A	1.017 (5)	0.861 (5)	0.322 (3)	0.041*	
H5A	0.694 (5)	−0.333 (4)	0.6256 (17)	0.041*	

### Atomic displacement parameters (Å^2^)

**Table:**

	*U*^11^	*U*^22^	*U*^33^	*U*^12^	*U*^13^	*U*^23^
O6	0.0764 (18)	0.0465 (14)	0.083 (2)	−0.0156 (12)	0.0381 (16)	−0.0248 (14)
Cu1	0.03280 (18)	0.02355 (17)	0.02061 (17)	−0.00261 (12)	−0.00096 (12)	0.00132 (11)
Cl1	0.0367 (3)	0.0334 (3)	0.0226 (3)	0.0066 (2)	0.0004 (2)	0.0039 (2)
N1	0.0277 (9)	0.0206 (9)	0.0236 (9)	−0.0004 (7)	0.0029 (7)	0.0005 (7)
N2	0.0319 (10)	0.0229 (9)	0.0229 (10)	−0.0007 (8)	0.0013 (8)	0.0011 (8)
N3	0.0386 (13)	0.0620 (18)	0.0352 (13)	0.0166 (12)	−0.0014 (10)	−0.0100 (12)
O1	0.0364 (10)	0.0346 (10)	0.0334 (10)	−0.0049 (8)	−0.0035 (8)	−0.0017 (8)
O2	0.0515 (12)	0.0230 (9)	0.0329 (10)	−0.0102 (8)	−0.0024 (9)	0.0017 (8)
O3	0.0758 (16)	0.0345 (11)	0.0312 (11)	−0.0132 (10)	−0.0119 (10)	0.0039 (9)
O4	0.0549 (13)	0.0326 (10)	0.0323 (10)	−0.0135 (9)	−0.0098 (9)	0.0055 (8)
O5	0.0518 (12)	0.0285 (9)	0.0276 (10)	−0.0093 (8)	−0.0032 (8)	0.0098 (8)
O7	0.0621 (13)	0.0303 (10)	0.0298 (10)	0.0002 (9)	−0.0052 (9)	−0.0007 (8)
O8	0.145 (3)	0.132 (3)	0.0376 (15)	0.086 (3)	−0.0179 (18)	−0.0050 (18)
C1	0.0498 (15)	0.0273 (12)	0.0242 (12)	−0.0046 (11)	−0.0033 (11)	0.0058 (10)
C2	0.0501 (16)	0.0250 (12)	0.0279 (13)	−0.0043 (11)	−0.0001 (11)	0.0078 (10)
C3	0.0278 (11)	0.0219 (11)	0.0267 (12)	−0.0015 (9)	0.0018 (9)	0.0004 (9)
C4	0.0298 (11)	0.0229 (11)	0.0219 (11)	−0.0017 (9)	0.0002 (9)	0.0032 (9)
C5	0.0269 (10)	0.0211 (10)	0.0216 (11)	0.0005 (8)	0.0042 (8)	0.0031 (8)
C6	0.0254 (10)	0.0204 (10)	0.0245 (11)	−0.0006 (8)	0.0049 (9)	0.0002 (8)
C7	0.0290 (11)	0.0202 (10)	0.0238 (11)	−0.0020 (8)	0.0023 (9)	0.0008 (8)
C8	0.0286 (11)	0.0207 (10)	0.0225 (11)	−0.0001 (8)	0.0038 (9)	0.0014 (8)
C9	0.0327 (12)	0.0205 (11)	0.0293 (12)	−0.0031 (9)	0.0036 (9)	0.0030 (9)
C10	0.0316 (12)	0.0213 (11)	0.0278 (12)	−0.0038 (9)	0.0020 (9)	0.0000 (9)
C11	0.0319 (12)	0.0235 (11)	0.0294 (12)	−0.0027 (9)	0.0027 (10)	0.0005 (9)
C12	0.0327 (12)	0.0216 (11)	0.0256 (11)	0.0002 (9)	0.0034 (9)	0.0034 (9)

### Geometric parameters (Å, °)

**Table:**

O6—N3	1.255 (4)	O5—C12	1.302 (3)
O6—Cu1	2.528 (3)	O5—H5A	0.790 (18)
Cu1—O1	1.982 (2)	C1—C2	1.387 (4)
Cu1—N1	1.999 (2)	C1—H1	0.93
Cu1—N2	2.002 (2)	C2—C3	1.378 (4)
Cu1—Cl1	2.2511 (7)	C2—H2	0.93
Cu1—Cl1^i^	2.7757 (8)	C3—C4	1.384 (3)
N1—C10	1.340 (3)	C3—C11	1.501 (3)
N1—C6	1.355 (3)	C4—C5	1.389 (3)
N2—C1	1.330 (3)	C4—H4	0.93
N2—C5	1.348 (3)	C5—C6	1.473 (3)
N3—O8	1.199 (4)	C6—C7	1.379 (3)
N3—O7	1.256 (3)	C7—C8	1.387 (3)
O1—H1WA	0.819 (18)	C7—H7	0.93
O1—H1WB	0.802 (18)	C8—C9	1.389 (3)
O2—C11	1.318 (3)	C8—C12	1.498 (3)
O2—H2A	0.77 (4)	C9—C10	1.384 (4)
O3—C11	1.196 (3)	C9—H9	0.93
O4—C12	1.207 (3)	C10—H10	0.93
			
N3—O6—Cu1	119.8 (2)	C2—C3—C4	119.9 (2)
O1—Cu1—N1	91.33 (8)	C2—C3—C11	121.1 (2)
O1—Cu1—N2	163.29 (9)	C4—C3—C11	119.0 (2)
N1—Cu1—N2	81.03 (8)	C3—C4—C5	118.5 (2)
O1—Cu1—Cl1	92.53 (6)	C3—C4—H4	120.8
N1—Cu1—Cl1	172.93 (6)	C5—C4—H4	120.8
N2—Cu1—Cl1	96.72 (6)	N2—C5—C4	121.5 (2)
O1—Cu1—O6	82.22 (9)	N2—C5—C6	114.76 (19)
N1—Cu1—O6	87.84 (10)	C4—C5—C6	123.8 (2)
N2—Cu1—O6	82.67 (9)	N1—C6—C7	121.6 (2)
Cl1—Cu1—O6	98.54 (8)	N1—C6—C5	114.4 (2)
C10—N1—C6	119.4 (2)	C7—C6—C5	124.0 (2)
C10—N1—Cu1	125.70 (16)	C6—C7—C8	118.9 (2)
C6—N1—Cu1	114.87 (16)	C6—C7—H7	120.5
C1—N2—C5	119.5 (2)	C8—C7—H7	120.5
C1—N2—Cu1	125.61 (17)	C7—C8—C9	119.5 (2)
C5—N2—Cu1	114.73 (16)	C7—C8—C12	118.5 (2)
O8—N3—O6	120.4 (3)	C9—C8—C12	122.0 (2)
O8—N3—O7	120.8 (3)	C10—C9—C8	118.6 (2)
O6—N3—O7	118.8 (3)	C10—C9—H9	120.7
Cu1—O1—H1WA	113 (3)	C8—C9—H9	120.7
Cu1—O1—H1WB	118 (3)	N1—C10—C9	121.9 (2)
H1WA—O1—H1WB	110 (4)	N1—C10—H10	119
C11—O2—H2A	106 (3)	C9—C10—H10	119
C12—O5—H5A	116 (3)	O3—C11—O2	124.3 (2)
N2—C1—C2	122.3 (2)	O3—C11—C3	123.3 (2)
N2—C1—H1	118.9	O2—C11—C3	112.3 (2)
C2—C1—H1	118.9	O4—C12—O5	124.2 (2)
C3—C2—C1	118.4 (2)	O4—C12—C8	122.1 (2)
C3—C2—H2	120.8	O5—C12—C8	113.7 (2)
C1—C2—H2	120.8		
			
N3—O6—Cu1—O1	−149.3 (3)	Cu1—N2—C5—C4	−174.51 (18)
N3—O6—Cu1—N1	119.1 (3)	C1—N2—C5—C6	−179.3 (2)
N3—O6—Cu1—N2	37.8 (3)	Cu1—N2—C5—C6	5.3 (3)
N3—O6—Cu1—Cl1	−57.9 (3)	C3—C4—C5—N2	−0.3 (4)
O1—Cu1—N1—C10	18.5 (2)	C3—C4—C5—C6	179.9 (2)
N2—Cu1—N1—C10	−176.4 (2)	C10—N1—C6—C7	−0.6 (3)
Cl1—Cu1—N1—C10	−104.5 (5)	Cu1—N1—C6—C7	−178.66 (18)
O6—Cu1—N1—C10	100.7 (2)	C10—N1—C6—C5	178.9 (2)
O1—Cu1—N1—C6	−163.57 (17)	Cu1—N1—C6—C5	0.9 (3)
N2—Cu1—N1—C6	1.50 (16)	N2—C5—C6—N1	−4.1 (3)
Cl1—Cu1—N1—C6	73.4 (5)	C4—C5—C6—N1	175.7 (2)
O6—Cu1—N1—C6	−81.41 (17)	N2—C5—C6—C7	175.4 (2)
O1—Cu1—N2—C1	−115.2 (3)	C4—C5—C6—C7	−4.7 (4)
N1—Cu1—N2—C1	−178.8 (2)	N1—C6—C7—C8	−0.1 (4)
Cl1—Cu1—N2—C1	7.9 (2)	C5—C6—C7—C8	−179.5 (2)
O6—Cu1—N2—C1	−89.9 (2)	C6—C7—C8—C9	0.3 (4)
O1—Cu1—N2—C5	59.8 (4)	C6—C7—C8—C12	179.4 (2)
N1—Cu1—N2—C5	−3.82 (17)	C7—C8—C9—C10	0.2 (4)
Cl1—Cu1—N2—C5	−177.06 (16)	C12—C8—C9—C10	−179.0 (2)
O6—Cu1—N2—C5	85.14 (19)	C6—N1—C10—C9	1.1 (4)
Cu1—O6—N3—O8	80.0 (4)	Cu1—N1—C10—C9	178.90 (19)
Cu1—O6—N3—O7	−100.2 (3)	C8—C9—C10—N1	−0.8 (4)
C5—N2—C1—C2	−0.5 (4)	C2—C3—C11—O3	−179.4 (3)
Cu1—N2—C1—C2	174.3 (2)	C4—C3—C11—O3	1.3 (4)
N2—C1—C2—C3	−0.4 (5)	C2—C3—C11—O2	2.1 (4)
C1—C2—C3—C4	1.0 (4)	C4—C3—C11—O2	−177.3 (2)
C1—C2—C3—C11	−178.3 (3)	C7—C8—C12—O4	−5.8 (4)
C2—C3—C4—C5	−0.6 (4)	C9—C8—C12—O4	173.4 (3)
C11—C3—C4—C5	178.7 (2)	C7—C8—C12—O5	174.0 (2)
C1—N2—C5—C4	0.8 (4)	C9—C8—C12—O5	−6.9 (4)

Symmetry codes: (i) −*x*+1, −*y*, −*z*.

### Hydrogen-bond geometry (Å, °)

**Table:**

*D*—H···*A*	*D*—H	H···*A*	*D*···*A*	*D*—H···*A*
C7—H7···O3^ii^	0.93	2.45	3.376 (3)	172
C4—H4···O3^ii^	0.93	2.42	3.346 (3)	179
C2—H2···Cl1^iii^	0.93	2.7	3.590 (3)	160
C1—H1···Cl1	0.93	2.67	3.258 (3)	122
O5—H5A···O7^iv^	0.790 (18)	1.798 (19)	2.582 (3)	172 (4)
O2—H2A···O4^ii^	0.77 (4)	1.92 (4)	2.676 (3)	169 (4)
O1—H1WB···O8^v^	0.802 (18)	2.10 (2)	2.830 (4)	152 (4)
O1—H1WA···Cl1^v^	0.819 (18)	2.48 (2)	3.220 (2)	152 (3)

Symmetry codes: (ii) −*x*+2, −*y*+1, −*z*+1; (iii) −*x*+1, −*y*+1, −*z*; (iv) −*x*+1, −*y*, −*z*+1; (v) −*x*, −*y*, −*z*.
